# Effects and Safety of FGF21 Analogs on Glycemic Parameters, Lipid Profiles, and Adiponectin in Overweight and Obese Adults: A Meta-Analysis of Randomized Controlled Trials

**DOI:** 10.1155/ije/9943228

**Published:** 2025-07-25

**Authors:** Zizheng Nie, Jiaoyang Xu, Yingying Liu, Qinglong Cao, Xinyi Qiu, Yiping Su, Shufen Han

**Affiliations:** ^1^School of Public Health and Nursing, Hangzhou Normal University, Hangzhou 311121, Zhejiang, China; ^2^Department of Dermatology, Hangzhou Medical College Affiliated Linan People's Hospital, The First People's Hospital of Hangzhou, Linan District, Hangzhou 311300, Zhejiang, China

**Keywords:** FGF21, glycemic parameters, lipid profiles, meta-analysis, obesity

## Abstract

**Objective:** Fibroblast growth factor 21 (FGF21) analogs have been used to improve glucose homeostasis and lipid metabolism; however, their effects remain contentious. The present meta-analysis aimed to review the effects and safety of FGF21 analogs on glycemic parameters, lipid profiles, and adiponectin (ADP) levels in overweight or obese adults.

**Methods:** A systematic literature search for randomized controlled trials (RCTs) was conducted up to June 2025. A random-effects model or a common-effect model was used to calculate the mean difference (MD) or standardized MD (SMD), along with the corresponding 95% confidence intervals (CIs).

**Results:** This meta-analysis including 11 RCTs showed that FGF21 analogs reduced triglycerides (MD = −59.33 mg/dL, 95% CI = −84.61 to −34.04), total cholesterol (MD = −17.14 mg/dL, 95% CI = −25.11 to −9.18), and low-density lipoprotein cholesterol (MD = −10.50 mg/dL, 95% CI = −14.42 to −6.59). Furthermore, FGF21 analogs increased high-density lipoprotein cholesterol (MD = 10.64 mg/dL, 95% CI = 6.23–15.05) and circulating ADP (MD = 3.18 μg/mL, 95% CI = 1.94–4.42). However, FGF21 analogs had no effect on fasting glucose (SMD = −0.22, 95% CI = −0.52 to 0.07) or insulin concentrations (SMD = −0.49, 95% CI = −1.04 to 0.06). Subgroup analyses revealed that the lipid-lowering effects varied among different FGF21 analogs. FGF21 treatment did not show any statistically significant difference in the incidence of serious side effects.

**Conclusions:** We identified significant favorable effects of FGF21 analogs in improving lipid profiles and elevating circulating ADP levels in overweight and obese adults. Future studies are needed to evaluate the clinical benefits in this area of research.

## 1. Introduction

Obesity is considered to be a global health priority due to its rising prevalence, with a nearly tripled rate from the 1970s to the present [1]. This surge is associated with a worldwide increase in the incidence of type 2 diabetes (T2DM), cardiovascular disease (CVD), and other metabolic disorders [2–4]. Obesity and its associated complications have significantly contributed to premature mortality in the general population [5, 6]. Recent investigations have demonstrated that disorders of glucose homeostasis and lipid metabolism result in the risk of obesity-related metabolic diseases [7]. Furthermore, the benefits of improving dysregulation extend beyond weight management and are related to a decreased risk of obesity-related metabolic complications.

**Figure 1:**
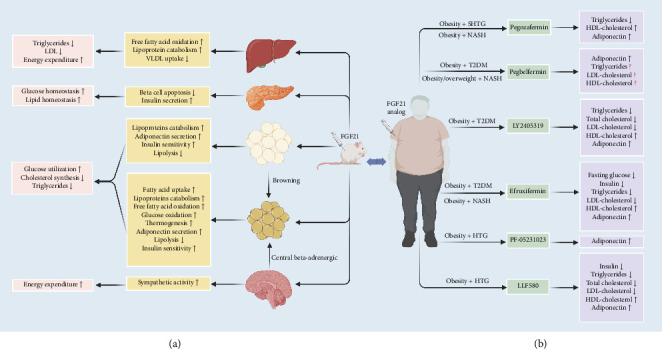
Potential health benefits of FGF21 on glucose homeostasis and lipid metabolism. (a) Animal studies suggest that FGF21 plays a differential regulatory role on glucose homeostasis and lipid metabolism across different target organs. (b) Clinical randomized controlled trials indicate that different FGF21 analogs present inconsistent effects on glycemic parameters, lipid profiles and circulating adiponectin in overweight and obese adults. LDL, low-density lipoprotein; VLDL, very low-density lipoproteins; HDL, high-density lipoprotein; HTG, hypertriglyceridemic; SHTG, severe hypertriglyceridemic; NASH, nonalcoholic steatohepatitis; T2DM, type 2 diabetes.

Fibroblast growth factor 21 (FGF21), an important stress-induced hormone, is synthesized by multiple organs and adipose tissue, and its production is enhanced in response to changes associated with metabolic states. As a novel metabolic regulator, FGF21 exerts its biological effects by a paracrine or endocrine manner in various target tissues and plays a crucial role in regulating glucose homeostasis and lipid metabolism [8, 9]. Initially, FGF21 was thought to possess glucose-lowering properties by increasing glucose uptake in adipocytes [10]. Recently, the beneficial effects of FGF21, which counteracts obesity and its related metabolic diseases by maintaining whole-body energy balance and protecting the liver from excessive triglycerides production and storage, have attracted considerable attention [11]. Animal studies revealed that FGF21 administration can stimulate thermogenesis and increase energy expenditure by accelerating triglyceride-rich lipoprotein turnover, improving insulin sensitivity, and strengthening browning of white fat [10, 12, 13] (Figure 1(a)). Considering its short half-life, endogenous FGF21 is not suitable for clinical application; therefore, long-acting FGF21 analogs have been used in preclinical trials for the treatment of obesity and associated metabolic disorders [11, 14, 15]. Several randomized controlled trials (RCTs) have assessed the effects of FGF21 analogs on glucose homeostasis and lipid metabolism in overweight and obese adults [16–19]. However, these results were inconsistent due to variations in the characteristics of the target population, differences in the chemical structure and dosages of FGF21 analogs, and the duration of treatment [16–26] (Figure 1(b)).

Therefore, we performed a systematic review and meta-analysis of RCTs to evaluate the effects of administering FGF21 analogs at appropriate doses on glycemic parameters, including fasting glucose and insulin, and lipid profiles, including triglycerides, total cholesterol, low-density lipoprotein (LDL) cholesterol, and high-DL (HDL) cholesterol, in overweight and obese adults. Additionally, adiponectin (ADP), as the most abundant peptide secreted by adipocytes, was also evaluated.

## 2. Materials and Methods

### 2.1. Search Strategy

This meta-analysis was conducted and reported according to the Preferred Reporting Items for Systematic Reviews and Meta-Analysis (PRISMA) guidelines (Table S1) [27]. The protocol was registered in the International Prospective Register of Systematic Reviews (https://www.crd.york.ac.uk/PROSPERO/), and the registration number was CRD42023380695. Relevant studies were systematically searched from PubMed, Cochrane Library, Scopus, and Web of Science up to June 2025, by using the following search terms: (FGF21) AND (Glucose OR insulin OR lipids OR cholesterol OR HDL OR LDL OR triglycerides OR ADP OR body weight). Detailed free terms based on the Medical Subject Headings (MeSH) were established for literature searches (Table S2). The reference lists of the retrieved articles were also screened to avoid missing studies.

### 2.2. Study Selection

Studies were searched based on the PICOS framework, which includes the participants, interventions, comparisons, outcomes, and study design (Table S3). Briefly, parallel RCTs involving overweight or obese adults were included in the present meta-analysis if they met the following criteria: (1) participants in the intervention groups received injections of FGF21 analogs, while participants in the control groups received placebo injections; (2) having an intervention duration that was at least two weeks; and (3) reporting the effects of FGF21 analogs on glycemic parameters, lipid profiles, circulating ADP, or body mass index (BMI), including net changes and their corresponding standard deviation (SD), or extracted data to calculate them. The exclusion criteria were as follows: (1) animal experiments or in vitro studies; (2) observational studies; (3) reviews or conference abstracts; and (4) studies that did not report the above-mentioned outcomes. Articles published in language other than English were excluded to avoid potential bias due to poor translation of information. Additionally, studies that examined the combined intervention of FGF21 with other drugs, utilizing alternative drugs as controls, were also excluded from the analysis.

### 2.3. Data Extraction

Two independent authors reviewed the literature search, data extraction, and quality assessment, with disagreements resolved by discussion with the third author. For each of the included RCTs, the following information was extracted: first author name; publication year and country; participant characteristics including sex, mean age, BMI and health status; study characteristics including study design, study duration, the number of participants in both intervention group and control group; the type and dosage of FGF21 analogs; the net changes in fasting glucose, insulin, triglycerides, total cholesterol, LDL-cholesterol, HDL-cholesterol, circulating ADP concentrations, or BMI and their corresponding SD; and adverse events. When a trial mentioned multiple intervention doses, the data on the most effective dose for improving glycemic parameters or lipid profiles were extracted according to the literature recommendation. If the authors did not offer a definitive recommendation, we determined the final effective dose based on a comprehensive evaluation and safety evaluation. In the absence of data, means and SD were calculated, or graphical information was retrieved using online tools (https://apps.automeris.io/wpd/index.zh_CN.html). The least squares means were not transformed and stability was verified by sensitivity analysis. When the units of these outcome indicators were inconsistent, we performed a unified unit conversion according to the literature report [28–30].

### 2.4. Quality Assessment

The quality of eligible RCTs was assessed using the Cochrane risk of bias (RoB) tool for evaluating the RoB by RevMan 5.4 software, which includes the following domains of bias: selection bias (random sequence generation; allocation concealment), performance bias (blinding of the participants and personnel), detection bias (blinding of outcome assessment), attrition bias (incomplete outcome data), reporting bias (selective outcome reporting), and other bias. Based on the mentioned items, each entry can be judged as “low risk”, “high risk”, or “unclear risk”, and the final overall quality of each RCT is considered to be good (low risk > 3 domains), fair (low risk = 3 domains), or poor (low risk < 3 domains) [31]. The Jadad scale was used to determine the quality of literature, which assesses the conduct of randomization, allocation concealment, blinding, and participant withdrawals or dropouts, with scores ranging from 0 to 7 [32]. A study with a scale rating of 4 or higher is evaluated as “high quality”; otherwise, it is deemed “low quality”.

### 2.5. Statistical Analysis

In this meta-analysis, the net changes for each indicator were calculated as the difference between the baseline and endpoint values in the intervention and control groups. For studies that did not report SD, the formula was utilized to calculate them based on standard errors or confidence intervals (CIs) [33]. For continuous data, the pooled effect was estimated using the mean difference (MD) when the units were consistent among studies; otherwise, the standardized MD (SMD) was used, and 95% CIs were also calculated. For categorical data, odds ratio (OR) with 95% CIs were used to summarize the effect size. The degree of heterogeneity across included studies was estimated using the Cochran *Q* test at the *p* < 0.10 level of significance and quantified by *I*^2^ statistics. *I*^2^ values < 25, 25%–75%, and > 75% were defined as low heterogeneity, moderate heterogeneity, and high heterogeneity, respectively [33]. In the presence of significant heterogeneity, a random-effects model was performed to calculate the pooled effect; otherwise, a common-effect model was used. To explore the contribution of an included RCTs to the overall MD, sensitivity analysis was performed by omitting one study each while recalculating the pooled effects for the remaining studies. Subgroup analyses of various FGF21 analogs were performed to obtain valuable insights into the effects of FGF21 on lipid profiles, circulating ADP, and adverse events. Potential publication bias was assessed using funnel plots and Egger's tests [34]. If publication bias was detected, the trim-and-fill method was used to correct the bias [35]. All analyses were performed using R software (version 4.2.3). *p* < 0.05 was considered statistically significant unless otherwise mentioned.

## 3. Results

### 3.1. Literature Search

**Figure 2:**
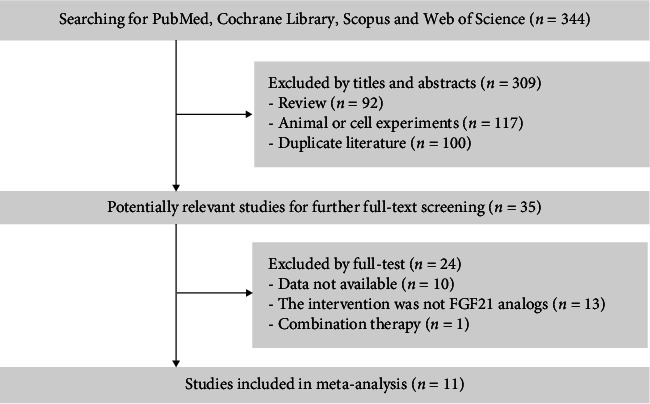
Flowchart of study selection.

The initial search found a total of 344 potentially relevant records, and 309 publications were excluded after checking the titles or abstracts. The full texts of the remaining 35 publications were reviewed, and 24 articles were excluded according to the inclusion criteria. Finally, a total of 11 RCTs were included in our meta-analysis [16–26]. In total, the eleven included publications reported the effects of FGF21 analogs on fasting glucose (*n* = 5), fasting insulin (*n* = 5), triglycerides (*n* = 9), total cholesterol (*n* = 2), LDL-cholesterol (*n* = 8), HDL-cholesterol (*n* = 9), and circulating ADP levels (*n* = 7). The flowchart of the selection process is shown in Figure 2.

### 3.2. Study Characteristics

The characteristics of the included trials are summarized in Table 1 and Table S4. The included literature was published from 2013 to 2024, and all the studies were conducted in the United States. The trials had a sample size ranging from 15 to 144, with a total of 312 participants in the FGF21 analogs treatment groups and 303 individuals in the control groups. A randomized and parallel design was followed in all trials. Six trials used triple blinding, and the remaining trials used double blinding (*n* = 5). During the study period, the study by Rader discontinued blinding due to side effects observed in the control groups; however, these adverse events were unrelated to the administration of FGF21 analogs [18]. The treatment duration lasted from 4 weeks to 48 weeks, with a median of 12 weeks. All trials included both women and men who were generally middle aged, with mean ages ranging from 45.5 to 60.2 years. Among the eleven included RCTs, the mean BMI of overweight and obese participants varied from 32.0 kg/m^2^ to 38.0 kg/m^2^. The participants were suffering from T2DM (*n* = 3), hypertriglyceridemia (*n* = 3) or nonalcoholic steatohepatitis (NASH, *n* = 5). Due to the diverse medical histories of the participants, one study required patients to receive atorvastatin, while another study discontinued the use of antidiabetic medications. Meanwhile, participants in other studies were taking metformin or statins. Included studies used pegbelfermin (*n* = 3), efruxifermin (*n* = 3), pegozafermin (*n* = 2), PF-05231023 (*n* = 1), LY2405319 (*n* = 1), LLF580 (*n* = 1), or AKR-001 (*n* = 1) as treatment arms. PF-05231023 was administered by intravenous injection, and other FGF21 analogs were given through subcutaneous injection. It is worth pointing out that FGF21 analogs were administered at different intervals: every four weeks (*n* = 1), weekly (*n* = 7), or daily (*n* = 3). Participants in the control group received an injection of either a placebo or 0.9% (w/v) sodium chloride.

### 3.3. Quality Assessment of Included Trials

**Figure 3:**
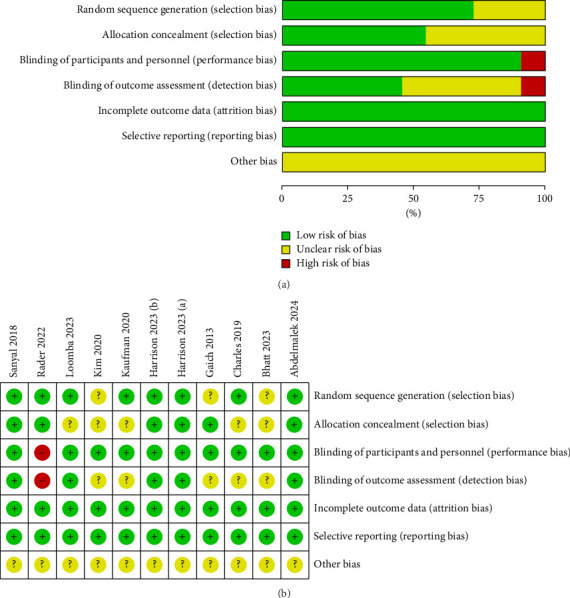
Risk of bias assessment for included randomized controlled trials. (a) Summary of the risk of bias assessment; (b) results of risk of bias assessment.

As shown in Figure 3 and Table S5, nine of the included RCTs were classified as good quality, and two RCTs were classified as fair quality according to the Cochrane RoB Assessment Tool. Briefly, three publications did not report the specific random sequence generation methods, five studies did not report the allocation concealment approaches, one study lacked blinding, and five studies did not mention blinding for outcome assessment. All studies had no selective reporting and provided complete outcome data. For the Jadad score, all studies had scores of at least four points (Table S6).

### 3.4. Effects of FGF21 Analogs on Glycemic Parameters

**Figure 4:**
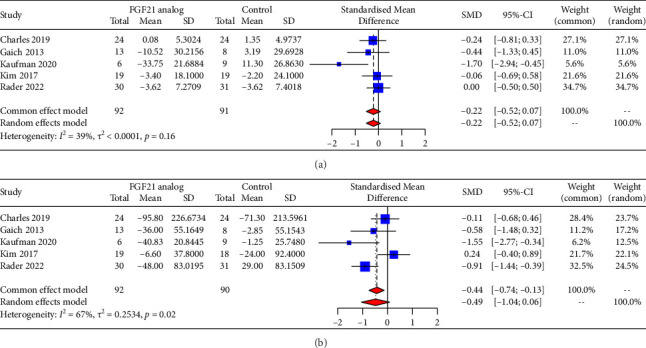
Forest plots for changes after administrating FGF21 analogs in glycemic parameters. (a) Fasting glucose; (b) fasting insulin. SMD, standardized mean difference.

Five RCTs were available for the analysis of FGF21 analogs on glycemic parameters. The net changes and corresponding 95% CIs for glycemic parameters in each trial and the overall estimate are shown in Figure 4. Compared with the control groups, FGF21 analogs produced a slight but not significant reduction in fasting glucose (SMD = −0.22, 95% CI = −0.52 to 0.07) with low heterogeneity (*I*^2^ = 39%) and fasting insulin concentrations (SMD = −0.49, 95% CI = −1.04 to 0.06) with moderate heterogeneity (*I*^2^ = 67%). To search for the sources of heterogeneity concerning FGF21 analogs on fasting insulin levels, sensitivity analysis was performed by omitting each trial. After excluding the trial conducted by Kim [21], FGF21 analogs resulted in a significant reduction in fasting insulin concentrations (SMD = −0.68, 95% CI = −1.22 to −0.14).

### 3.5. Effects of FGF21 Analogs on Lipid Profiles

**Figure 5:**
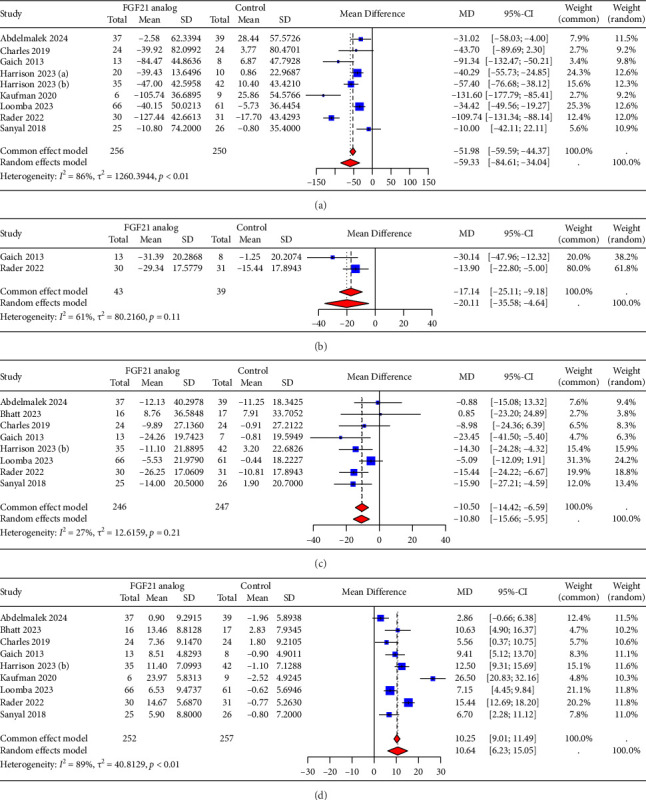
Forest plots for changes after administrating FGF21 analogs in lipid profiles. (a) Triglycerides; (b) total cholesterol; (c) low-density lipoprotein cholesterol; (d) high-density lipoprotein cholesterol. MD, mean difference.

For lipid profiles, nine trials were available for the analysis of FGF21 analogs on triglycerides and HDL-cholesterol, two on total cholesterol, and eight on LDL-cholesterol. Compared with the control groups, FGF21 analogs demonstrated a significant reduction in triglycerides (−59.33 mg/dL, 95% CI = −84.61 to −34.04, Figure 5(a)), total cholesterol (−17.14 mg/dL, 95% CI = −25.11 to −9.18, Figure 5(b)), and LDL-cholesterol (−10.50 mg/dL, 95% CI = −14.42 to −6.59, Figure 5(c)), and resulted in a marked increase in HDL-cholesterol (10.64 mg/dL, 95% CI = 6.23 to 15.05, Figure 5(d)). No heterogeneity was observed in the analysis of LDL-cholesterol (*I*^2^ = 27%), whereas moderate to high heterogeneity was observed in the analysis of triglycerides (*I*^2^ = 86%), total cholesterol (*I*^2^ = 61%), and HDL-cholesterol (*I*^2^ = 89%). For triglycerides and HDL-cholesterol, sensitivity analyses showed that none of the trials could substantially influence the pooled effect, with triglycerides ranging from −51.70 mg/dL (95% CI = −75.07 to −28.33) to −65.15 mg/dL (95% CI = −90.58 to −39.72), and HDL-cholesterol ranging from 8.89 mg/dL (95% CI = 5.90–11.87) to 11.64 mg/dL (95% CI = 7.16–16.12). Subgroup analyses of various FGF21 analogs indicated significant differences in the reduction of triglycerides levels (Figure S1, *p* < 0.01) and the increase of HDL-cholesterol levels (Figure S3, *p* < 0.01). However, no significant differences were detected among subgroups concerning the reduction of LDL-cholesterol levels (Figure S2, *p*=0.15). The administration of efruxifermin resulted in a greater reduction in TG of 72.41 mg/dL and a more significant increase in HDL-cholesterol of 19.29 mg/dL, while pegbelfermin caused a lesser decrease in TG of 25.90 mg/dL and a smaller increase in HDL-cholesterol of 4.62 mg/dL.

### 3.6. Effect of FGF21 Analogs on Circulating ADP and BMI

**Figure 6:**
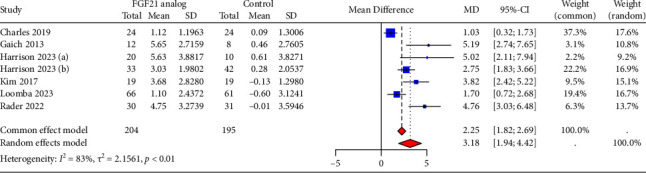
Forest plot for changes after administrating FGF21 analogs in circulating adiponectin levels. MD, mean difference.

The net change and the corresponding 95% CI for circulating ADP levels for 7 RCTs are presented in Figure 6. Considering the evidence of heterogeneity (*I*^2^ = 83%), a random-effects model was used, and the administration of FGF21 analogs resulted in a significant increase in circulating ADP levels (3.18 μg/mL, 95% CI = 1.94–4.42). Sensitivity analysis revealed that none of the trials had a significant impact on the overall pooled effect, with ADP levels ranging from 2.91 μg/mL (95% CI = 1.62–4.21) to 3.56 mg/dL (95% CI = 2.40–4.73). Subgroup analyses indicated that various FGF21 analogs exhibit varying effects on enhancing circulating ADP levels (*p* < 0.01, Figure S4). Additionally, a pooled analysis of five eligible RCTs found a significant reduction in BMI with FGF21 analogs treatment, with a MD of −0.39 kg/m^2^ (95% CI = −0.58 to −0.20, Figure S5) and no observed heterogeneity (*I*^2^ = 0%).

### 3.7. Publication Bias

**Figure 7:**
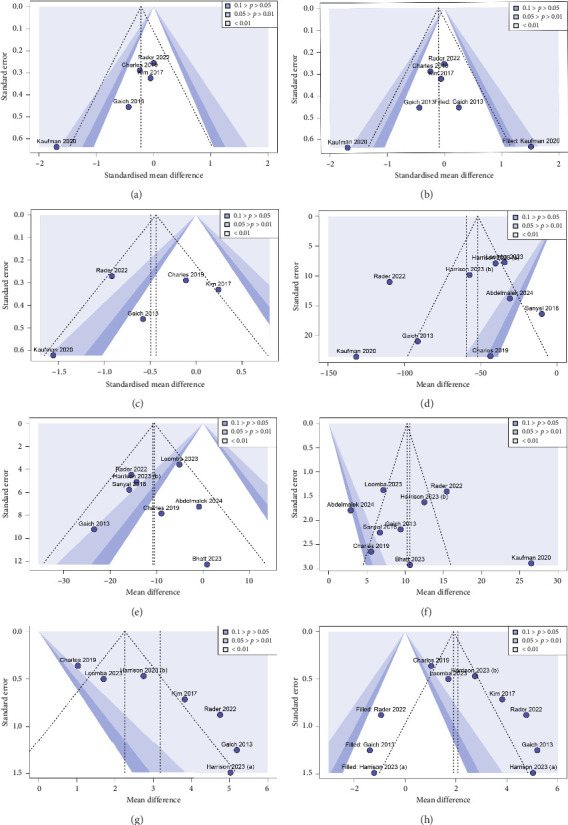
Funnel plots for publication bias. (a) Fasting glucose; (b) fasting glucose adjustment by trim-and-fill method; (c) fasting insulin; (d) triglycerides; (e) low-density lipoprotein cholesterol; (f) high-density lipoprotein cholesterol; (g) adiponectin; (h) adiponectin adjustment by trim-and-fill method.

Funnel plots and Egger's test were used to assess publication bias (Figure 7). The visual observation of the funnel plots and Egger's test results indicated the presence of potential publication bias for fasting glucose and ADP. We used the trim-and-fill method to adjust for potential publication bias. The combined effect for fasting glucose remained relatively stable (SMD = −0.096, 95% CI: −0.55 to 0.36). A significant change in circulating ADP was found following the trim-and-fill adjustment (MD = 2.082, 95% CI = 1.97–3.39); however, sensitivity analyses showed that the results were robust and that publication bias did not affect the pooled effect. Egger's test showed no significant publication bias for fasting insulin (*p*=0.587), triglycerides (*p*=0.390), LDL-cholesterol (*p*=0.860) and HDL-cholesterol (*p*=0.787).

### 3.8. Adverse Events

Most of the included RCTs reported that the administration of FGF21 analogs led to mild side effects, with nausea and diarrhea being the most frequently observed adverse effects (Table S7). The frequency of serious adverse events in the FGF21 groups was not significantly different from that in the control groups (OR = 1.72, 95% CI = 0.81 to 3.68, *I*^2^ = 0%, Figure S6). Subgroup analyses revealed no significant differences in serious adverse reactions across the various FGF21 analogs (*p* = 0.89, Figure S7).

## 4. Discussion

To the best of our knowledge, this is the first systematic review and meta-analysis of RCTs examining the effects and safety of FGF21 analogs on glycemic parameters, lipid profiles, and circulating ADP levels in overweight and obese adults. Our results suggested that the administration of FGF21 analogs resulted in significant reductions in triglycerides (59.33 mg/dL), total cholesterol (17.14 mg/dL), and LDL-cholesterol (10.50 mg/dL), but marked elevations in HDL-cholesterol (10.64 mg/dL) and circulating ADP levels (3.18 μg/mL) in overweight and obese adults. FGF21 analogs did not significantly affect the concentrations of fasting glucose and fasting insulin. Additionally, the administration of FGF21 analogs was associated with a significant reduction in BMI, suggesting potential benefits for weight reduction in this population. Subgroup analyses indicated that the administration of efruxifermin produced a more pronounced lipid-lowering effect, as evidenced by a reduction in TG and an increase in HDL-cholesterol. The included studies reported that the administration of FGF21 analogs caused mild gastrointestinal reactions. No significant differences in serious adverse events were observed between the two groups receiving FGF21 analogs and the placebo.

The present meta-analysis attempts to verify that the methodological quality assessment meets the standardized procedures in this field by following the PRISMA guidelines and using the RoB assessment and the Jadad score. Our meta-analysis was regarded as a relatively high-quality study, as all Jadad scores were no less than four points. Nevertheless, we should be more cautious in interpreting the present findings because of considerable heterogeneity among the trials. Only well-designed RCTs with a large sample size were enrolled to ensure the validity of the results.

Our results suggested that FGF21 analogs had no effect on fasting glucose and insulin levels in overweight and obese adults. However, the therapeutic administration of FGF21 has demonstrated a glucose-lowering effect, along with an enhancement of insulin sensitivity in diabetic mice [10]. In our included studies, some participants received FGF21 analogs along with other medications, such as metformin, and the baseline fasting glucose levels were not high [19]. Consequently, the glucose-lowering effect was difficult to observe. Although there was no significant reduction in fasting insulin levels after FGF21 analog treatment, sensitivity analyses showed that the result was dependent on the study by Kim [21], which accounted for a weight of 22.1%. When the study was removed, the administration of FGF21 analogs resulted in a statistically significant reduction in fasting insulin levels. This study conducted by Kim used PF-05231023 as a treatment arm, which had no effect on fasting insulin levels [21]. Considering the variations in pharmacokinetic properties of different FGF21 analogs, the effect of FGF21 in improving insulin sensitivity is based on sustained exposure to the intact C-terminus [21, 36]. Despite the structural and functional similarity of FGF21 analogs, there may be variations in their synthesis methods and pathways. PF-05231023 is recognized as a functionally active and potent FGF21 agonist [36], while efruxifermin is classified as a long-acting fusion protein [17]. Early animal studies suggested that FGF21, recognized as an effective insulin-sensitizing agent, may alleviate obesity-associated hyperglycemia by enhancing insulin sensitivity of hepatic and adipose tissues [10, 37, 38]; however, FGF21 levels were paradoxically elevated in insulin-resistance states [39], potentially due to either a resistance to its function or a compensatory increase in its secretion [40]. Additionally, ADP is obligatory for the glucose-lowering and insulin-sensitizing effects of FGF21, as it couples FGF21 actions in local adipocytes to liver and skeletal muscle [41]. Consequently, further research is needed to explore the potential effects of FGF21 on glycemic parameters by using a larger sample size, extending the duration of treatment, ensuring consistent patient characteristics (such as comparable baseline glucose levels and medication backgrounds), and employing a distinct FGF21 analog.

Hyperlipidemia is a common metabolic complication for overweight and obese adults. Findings from our meta-analysis suggested that the administration of FGF21 analogs was beneficial for improving lipid profiles and may be helpful in regulating lipid metabolism and enhancing lipid deposition in overweight and obese adults. Although there was considerable heterogeneity in triglycerides, total cholesterol and HDL-cholesterol levels, sensitivity analyses demonstrated that the results remained robust after omitting one study each. Furthermore, subgroup analyses indicated that different FGF21 analogs exhibited varying lipid-lowering effects. The differences may be attributed to the chemical structural variations among the FGF21 analogs, as well as the therapeutic doses administered. Additionally, the sample size and the number of studies included in subgroup analyses were relatively small. It is worth mentioning that efruxifermin demonstrated greater efficacy than pegbelfermin in lowering triglycerides levels and increasing HDL-cholesterol levels. This increased effectiveness may be attributed to the longer half-life of efruxifermin and its ability to interact with multiple receptors, which collectively enhancing its lipid-lowering effects [42, 43]. The lipid-lowering effects of various FGF21 analogs necessitate further validation through larger and longer-term RCTs. The lipid-lowering effect of FGF21 analogs may be directly attributed to the inhibition of hepatic de novo lipogenesis and the reduction of hepatic lipotoxicity [44]. Alternatively, this effect may be indirectly mediated by ADP, which reduces hepatic steatosis and inflammation [45]. Furthermore, studies conducted in both animals and humans have shown that FGF21can promote adipose tissue lipolysis and reduce body weight [46], which may be one of the mechanisms by which FGF21 lowers blood lipids. Our result also demonstrated that the administration of FGF21 analogs significantly reduced BMI in overweight and obese adults. This finding further supports to the notion that the lipid-lowering effects of FGF21 analogs may be partially mediated through facilitating weight reduction. The present meta-analysis and subgroup analyses revealed that the administration of FGF21 analogs increased circulating ADP levels. Studies have demonstrated that the regulation of glucose homeostasis and lipid metabolism by FGF21 is based on circulating ADP secretion in rodent models [38, 47]. In adipose tissues of obese mice, impaired FGF21 signaling cascades led to a defect in the ability of FGF21 to induce GLUT1-dependent glucose uptake and ADP secretion, thereby resulting in insulin resistance [8, 48], while FGF21 infusion could improve glucose uptake and insulin sensitivity in obese mice [37].

FGF21, a recently recognized metabolic regulator of glucose and lipid metabolism as well as energy homeostasis [49], has gained significant attention as a promising therapeutic agent for metabolic disorders, including NASH, T2DM, and obesity [50–53]. FGF21 plays a metabolic regulation role by binding to FGF receptors located on target organs, such as adipose tissue, liver, and muscle [54]. It is widely believed that the mechanism by which FGF21 improves metabolic disorders is associated with its unique protein-protein interaction and crosstalk with peroxisome proliferator-activated receptor [50]. Their combined role collectively contributes to the inhibition of inflammation, the reduction of oxidative stress and, and the enhancement of lipid catabolism. Furthermore, FGF21 has been shown to strongly stimulate the secretion of ADP [55], a crucial regulator of metabolic homeostasis [56]. Our meta-analysis demonstrated that the administration of FGF21 analogs can significantly lower blood lipid levels and increase circulating ADP levels in overweight and obese adults. Concomitantly, there are safety concerns about the side effects of FGF21 analogs in potentially long-term treatment. The present findings revealed that nausea and diarrhea were the most common adverse events reported following the ingestion of FGF21 analogs. The administration of FGF21 analogs showed no statistically significant difference in serious side effects compared to the control groups. Therefore, FGF21 analogs were generally safe and well tolerated in the population study.

The present meta-analysis has several limitations and drawbacks, which may warrant further investigation through a large, long-term, well-designed RCT or a multicenter collaborative RCT. First, due to the lack of consistent FGF21 analogs treatment, different FGF21 analogs may have different effects on glycemic parameters and lipid profiles. This also limits to analyze the dose-response effect of FGF21 analogs on glycemic parameters, lipid profiles, and ADP levels. Given the complexity of the structure and the functional difference of FGF21 analogs, more studies are warranted using the same FGF analogs in future RCTs to confirm our findings. Second, the limited number of included RCTs and small sample sizes limited our ability to assess the potential sources of heterogeneity by performing comprehensive subgroup analyses. The heterogeneity observed among the studies may be attributed to varying doses or durations of FGF21 analogs, as well as the health status and medication use of overweight and obese adults. It is noteworthy that lifestyle factors (e.g., physical activity and dietary habits) are possible confounders affecting the results of this meta-analysis. Given these considerations, the current results should be interpreted with caution. Third, the possible biases regarding the treatment effects cannot be fully ruled out, as most of the included RCTs failed to provide adequate relevant information for the assessment of potential RoB. Furthermore, we extracted BMI values from only five of the included RCTs; therefore, the effects of FGF21 analogs on weight management require further investigation in future studies. Finally, the baseline data of glycemic parameters, lipid profiles, and circulating ADP levels in these included RCTs differed and may be influenced asynchronously during FGF21 analog treatment. Large-scale clinical trials using high-quality randomized designs—featuring comparable baseline glucose and lipid levels, as well as consistent medication backgrounds—are needed to further confirm the clinical benefits of FGF21 analogs on glycemic parameters, lipid profiles, and ADP in overweight and obese adults.

## 5. Conclusion

Based on the current evidence, this meta-analysis supports the beneficial effects of administering FGF21 analogs on lipid profiles and circulating ADP levels in overweight and obese adults, and indicates that the use of FGF21 analogs is relatively safe in this population. Furthermore, different types of FGF21 analogs exhibit varying benefits in improving lipid profiles and circulating ADP levels. However, the present study found no evidence to support the efficacy of FGF21 analogs in reducing glycemic parameters. Considering the existence of moderate or high heterogeneity in partial indicators, the results should be interpreted with caution. A large, long-term, well-designed RCT or a multicenter collaborative RCT is warranted to provide deeper insights into the clinical benefits of FGF21 analogs on glycemic parameters, lipid profiles, and circulating ADP levels in different metabolic disorders.

## Figures and Tables

**Table 1:** Characteristics of the trials and participants in this meta-analysis.

First author (year)	Study design	Size^a^	Male (%)	Age (year)	Study period (weeks)	BMI (kg/m^2^)	FGF21	Baseline^a^							Population health	Jadad scores
								Glucose	Insulin	TG	TC	LDL-C	HDL-C	ADP		
Abdelmalek (2024)	P, TB	37/39	35.5	60.2	48	35.4	Pegbelfermin 20 mg/week	—	—	158/133	—	99/93	47/50	—	NASH and compensated cirrhosis	7
Bhatt (2023)	P, DB	18/18	69.4	55.7	8	33.7	Pegozafermin 27 mg/week	158/124	—	645/575	234/248	97/88	31/28	4.9/4.0	Severe HTG	4
Charles (2019)	P, DB	24/24	60.5	57.0	12	35.0	Pegbelfermin 20 mg/day	151/160	138/115	191/193	—	89/102	48/50	3.1/3.5	T2DM	5
Gaich (2013)	P, DB	15/10	48.0	57.6	4	32.0	LY2405319 20 mg/day	186/162	88/66	189/277	204/199	120/107	44/39	6.8/4.2	T2DM	6
Harrison (2023)	P, TB	20/10	36.7	59.7	16	37.0	Efruxifermin 50 mg/week	107/123	201/264	135/122	167/157	90/90	50/43	5.8/4.8	Compensated NASH cirrhosis	7
Harrison (2023)	P, TB	43/43	41.9	53.7	24	38.0	Efruxifermin 50 mg/week	—	—	154/170	—	111/94	41/42	3.5/3.4	NASH	7
Kaufman (2020)	P, DB	6/9	53.3	54.2	4	32.4	Efruxifermin 70 mg/week	190/163	93/61	177/142	197/201	—	42/46	5.2/3.3	T2DM	5
Kim (2017)	P, DB	21/22	65.1	53.0	4	33.7	PF-05231023 150 mg/week	—	—	206/224	154/153	89/84	43/44	—	HTG	4
Loomba (2023)	P, TB	73/71	38.4	55.8	24	36.6	Pegozafermin 30 mg/week	—	—	175/170	—	—	—	—	NASH	5
Rader (2022)	P, TB	30/31	49.2	45.5	12	36.1	LLF580 300 mg/4 weeks	101/101	148/156	225/226	216/212	145/143	45/42	5.0/4.3	HTG	5
Sanyal (2018)	P, TB	25/26	39.2	49.5	16	35.5	Pegbelfermin 10 mg/day	—	—	208/171	—	129/128	47/50	—	NASH	7

*Note:* P, parallel; glucose, fasting glucose (mg/dL); insulin, fasting insulin (pmol/L); TG, triglycerides (mg/dL); ADP, adiponectin (μg/mL); T2DM, type 2 diabetes; NASH, nonalcoholic steatohepatitis; HTG, hypertriglyceridemic; “—”, not reported.

Abbreviations: DB, double-blind; HDL-C, high-density lipoprotein cholesterol (mg/dL); LDL-C, low-density lipoprotein cholesterol (mg/dL); TB, triple-blind; TC, total cholesterol (mg/dL).

^a^The sample size and the baseline of outcomes were expressed as intervention group/control group.

## Data Availability

The data used to support the findings of this study are included within the article.

## Supporting Information

Table S1: PRIMA Checklist for the meta-analysis.

Table S2: Search strategy.

Table S3: Participants, interventions, comparisons, outcomes, and study design.

Table S4: Detailed information of trials in this meta-analysis.

Table S5: The methodological quality of the included RCTs via the risk of bias.

Table S6: The methodological quality of the included RCTs via the modified Jadad scale.

Table S7: Safety summary.

Figure S1: Subgroup analyses for different FGF21 analogs in triglycerides. MD, mean difference.

Figure S2: Subgroup analyses for different FGF21 analogs in low-density lipoprotein cholesterol. MD, mean difference.

Figure S3: Subgroup analyses for various FGF21 analogs in high-density lipoprotein cholesterol. MD, mean difference.

Figure S4: Subgroup analyses for different FGF21 analogs in circulating adiponectin levels. MD, mean difference.

Figure S5: Forest plots for changes after administrating FGF21 analogs in body mass index. MD, mean difference.

Figure S6: Forest plot for incidence of serious adverse events after administrating FGF21 analogs. OR, odds ratio.

Figure S7: Subgroup analyses for various FGF21 analogs in incidence of serious adverse events. OR, odds ratio.
