# Supplementary material for: Effects and Safety of FGF21 Analogs on Glycemic Parameters, Lipid Profiles, and Adiponectin in Overweight and Obese Adults: A Meta-Analysis of Randomized Controlled Trials
Source: Int J Endocrinol. 2025 Jul 25;2025:9943228. doi: 10.1155/ije/9943228 (PMC12316501; doi:10.1155/ije/9943228)
Supplement: Supporting Information — Additional supporting information can be found online in the Supporting Information section. [file 9943228.f1.docx]

**Table S1.** PRIMA Checklist for the meta-analysis.

| **cSection and Topic** | **Item #** | **Checklist item** | **Location where item is reported** |
| --- | --- | --- | --- |
| **TITLE** | | |  |
| Title | 1 | Identify the report as a systematic review. | Title page |
| **ABSTRACT** | | |  |
| Abstract | 2 | See the PRISMA 2020 for Abstracts checklist. | Abstract |
| **INTRODUCTION** | | |  |
| Rationale | 3 | Describe the rationale for the review in the context of existing knowledge. | Introduction: paragraph 1-2;  Figure 1 |
| Objectives | 4 | Provide an explicit statement of the objective(s) or question(s) the review addresses. | Introduction: paragraph 3;  Supplementary table 3 |
| **METHODS** | | |  |
| Eligibility criteria | 5 | Specify the inclusion and exclusion criteria for the review and how studies were grouped for the syntheses. | Materials and methods: study selection |
| Information sources | 6 | Specify all databases, registers, websites, organisations, reference lists and other sources searched or consulted to identify studies. Specify the date when each source was last searched or consulted. | Materials and methods: search strategy |
| Search strategy | 7 | Present the full search strategies for all databases, registers and websites, including any filters and limits used. | Materials and methods: study strategy;  Supplementary table 2 |
| Selection process | 8 | Specify the methods used to decide whether a study met the inclusion criteria of the review, including how many reviewers screened each record and each report retrieved, whether they worked independently, and if applicable, details of automation tools used in the process. | Materials and methods: study selection and data extraction;  Figure 2 |
| Data collection process | 9 | Specify the methods used to collect data from reports, including how many reviewers collected data from each report, whether they worked independently, any processes for obtaining or confirming data from study investigators, and if applicable, details of automation tools used in the process. | Materials and methods: data extraction |
| Data items | 10a | List and define all outcomes for which data were sought. Specify whether all results that were compatible with each outcome domain in each study were sought (e.g. for all measures, time points, analyses), and if not, the methods used to decide which results to collect. | Materials and methods: data extraction |
|  | 10b | List and define all other variables for which data were sought (e.g. participant and intervention characteristics, funding sources). Describe any assumptions made about any missing or unclear information. | Materials and methods: data extraction |
| Study risk of bias assessment | 11 | Specify the methods used to assess risk of bias in the included studies, including details of the tool(s) used, how many reviewers assessed each study and whether they worked independently, and if applicable, details of automation tools used in the process. | Materials and methods: quality assessment |
| Effect measures | 12 | Specify for each outcome the effect measure(s) (e.g. risk ratio, mean difference) used in the synthesis or presentation of results. | Materials and methods: statistical analysis |
| Synthesis methods | 13a | Describe the processes used to decide which studies were eligible for each synthesis (e.g. tabulating the study intervention characteristics and comparing against the planned groups for each synthesis (item #5)). | Materials and methods: study selection;  Figure 2;  Table 1;  Supplementary table 4 |
|  | 13b | Describe any methods required to prepare the data for presentation or synthesis, such as handling of missing summary statistics, or data conversions. | Materials and methods: data extraction and statistical analysis |
|  | 13c | Describe any methods used to tabulate or visually display results of individual studies and syntheses. | Materials and methods: statistical analysis |
|  | 13d | Describe any methods used to synthesize results and provide a rationale for the choice(s). If meta-analysis was performed, describe the model(s), method(s) to identify the presence and extent of statistical heterogeneity, and software package(s) used. | Materials and methods: statistical analysis |
|  | 13e | Describe any methods used to explore possible causes of heterogeneity among study results (e.g. subgroup analysis, meta-regression). | Materials and methods: statistical analysis |
|  | 13f | Describe any sensitivity analyses conducted to assess robustness of the synthesized results. | Materials and methods: statistical analysis |
| Reporting bias assessment | 14 | Describe any methods used to assess risk of bias due to missing results in a synthesis (arising from reporting biases). | Materials and methods: quality assessment |
| Certainty assessment | 15 | Describe any methods used to assess certainty (or confidence) in the body of evidence for an outcome. | Materials and methods: statistical analysis |
| **RESULTS** | | |  |
| Study selection | 16a | Describe the results of the search and selection process, from the number of records identified in the search to the number of studies included in the review, ideally using a flow diagram. | Results: literature search;  Figure 2 |
|  | 16b | Cite studies that might appear to meet the inclusion criteria, but which were excluded, and explain why they were excluded. | Results: literature search |
| Study characteristics | 17 | Cite each included study and present its characteristics. | Results: study characteristics,  Table 1;  Supplementary table 4 |
| Risk of bias in studies | 18 | Present assessments of risk of bias for each included study. | Results: quality assessment of included trials;  Figure 3;  Supplementary table 5-6 |
| Results of individual studies | 19 | For all outcomes, present, for each study: (a) summary statistics for each group (where appropriate) and (b) an effect estimate and its precision (e.g. confidence/credible interval), ideally using structured tables or plots. | Figure 4·6;  Supplementary figure 5-6 |
| Results of syntheses | 20a | For each synthesis, briefly summarise the characteristics and risk of bias among contributing studies. | Results: effects of FGF21 analogs on glycemic parameters, effects of FGF21 analogs on lipid profiles, effect of FGF21 analogs on circulating ADP and BMI and adverse events |
|  | 20b | Present results of all statistical syntheses conducted. If meta-analysis was done, present for each the summary estimate and its precision (e.g. confidence/credible interval) and measures of statistical heterogeneity. If comparing groups, describe the direction of the effect. | Results: effects of FGF21 analogs on glycemic parameters, effects of FGF21 analogs on lipid profiles, effect of FGF21 analogs on circulating ADP and BMI and adverse events;  Supplementary figure 1-4 |
|  | 20c | Present results of all investigations of possible causes of heterogeneity among study results. | Results: effects of FGF21 analogs on glycemic parameters, effects of FGF21 analogs on lipid profiles, effect of FGF21 analogs on circulating ADP and BMI and adverse events |
|  | 20d | Present results of all sensitivity analyses conducted to assess the robustness of the synthesized results. | Results: effects of FGF21 analogs on glycemic parameters, effects of FGF21 analogs on lipid profiles, effect of FGF21 analogs on circulating ADP and BMI and adverse events |
| Reporting biases | 21 | Present assessments of risk of bias due to missing results (arising from reporting biases) for each synthesis assessed. | Results: quality assessment of included trials;  Figure 3;  Supplementary table 5-6 |
| Certainty of evidence | 22 | Present assessments of certainty (or confidence) in the body of evidence for each outcome assessed. | Results: quality assessment of included trials and publication bias |
| **DISCUSSION** | | |  |
| Discussion | 23a | Provide a general interpretation of the results in the context of other evidence. | Discussion: paragraph 1 |
|  | 23b | Discuss any limitations of the evidence included in the review. | Discussion: paragraph 2 |
|  | 23c | Discuss any limitations of the review processes used. | Discussion: paragraph 6 |
|  | 23d | Discuss implications of the results for practice, policy, and future research. | Discussion: paragraph 5 |
| **OTHER INFORMATION** | | |  |
| Registration and protocol | 24a | Provide registration information for the review, including register name and registration number, or state that the review was not registered. | CRD42023380695 |
|  | 24b | Indicate where the review protocol can be accessed, or state that a protocol was not prepared. | https://www.crd.york.ac.uk/PROSPERO/ |
|  | 24c | Describe and explain any amendments to information provided at registration or in the protocol. | N/A |
| Support | 25 | Describe sources of financial or non-financial support for the review, and the role of the funders or sponsors in the review. | Funding |
| Competing interests | 26 | Declare any competing interests of review authors. | Declaration of interest |
| Availability of data, code and other materials | 27 | Report which of the following are publicly available and where they can be found: template data collection forms; data extracted from included studies; data used for all analyses; analytic code; any other materials used in the review. | Data availability |

**Table S2.** Search strategy.

| **Medical Subject Headings (MeSH)** | **Free-word** |
| --- | --- |
| #1 fibroblast growth factor 21 | FGF-21  FGF21 |
| #2 Glucose | D-Glucose  D Glucose  Dextrose  Glucose, (alpha-D)-Isomer  Anhydrous Dextrose  Dextrose, Anhydrous  Glucose, (DL)-Isomer  Glucose, (L)-Isomer  L-Glucose  L Glucose  Glucose Monohydrate  Monohydrate, Glucose  Glucose, (beta-D)-Isomer |
| #3 Insulin | Insulin, Regular  Regular Insulin  Soluble Insulin  Insulin, Soluble  Insulin A Chain  Sodium Insulin  Insulin, Sodium  Novolin  Iletin  Insulin B Chain  Chain, Insulin B |
| #4 Cholesterol, LDL | Low Density Lipoprotein Cholesterol  beta-Lipoprotein Cholesterol  Cholesterol, beta-Lipoprotein  beta Lipoprotein Cholesterol  LDL Cholesterol  Cholesteryl Linoleate, LDL  LDL Cholesteryl Linoleate |
| #5 Cholesterol, HDL | alpha-Lipoprotein Cholesterol  Cholesterol, alpha-Lipoprotein  alpha Lipoprotein Cholesterol  HDL Cholesterol  High Density Lipoprotein Cholesterol  Cholesterol, HDL2  HDL2 Cholesterol  HDL(2) Cholesterol  Cholesterol, HDL3  HDL3 Cholesterol  HDL(3) Cholesterol |
| #6 Triglycerides | Triacylglycerols  Triacylglycerol  Triglyceride |
| #7 Adiponectin | Adipocyte Complement-Related Protein 30-kDa  Adipocyte Complement Related Protein 30 kDa  Adipose Most Abundant Gene Transcript 1  apM-1 Protein  apM 1 Protein  ACRP30 Protein  Adipocyte, C1q and Collagen Domain Containing Protein |
| #8 Body weight | Body Weights  Weight, Body  Weights, Body |

***Method:*** #1 AND (#2 OR #3 OR #4 OR #5 OR #6 OR #7 OR #8)

**Table S3** Participants, interventions, comparisons, outcomes, and study design.

| **Parameter** | **Criteria** |
| --- | --- |
| Participants | Overweight or obese adults (BMI ≥ 25kg/m^2^) |
| Interventions | FGF21 analogs by injection for a duration not less than 2 weeks |
| Comparisons | Receiving placebo or control or usual care |
| Outcomes | Fasting glucose, insulin, triglycerides, total cholesterol, low-density lipoprotein cholesterol, high-density lipoprotein cholesterol, adiponectin and adverse events |
| Study design | Parallel randomized controlled trials |

***Note:*** BMI, body mass index.

| **First author (Year, country)** | **Size**  **(Withdraw)** | **Analysis** | **Intervention methods** | **Results** | |
| --- | --- | --- | --- | --- | --- |
|  |  |  |  | **Significant effect** | **No effect** |
| Abdelmalek  (2024, USA/Japan) | T: 37 (6)  C: 39 (5) | T: 37  C: 39 | T: Pegbelfermin 20mg QW, SC  C: Placebo QW, SC | - | Triglycerides, HDL-cholesterol, LDL-cholesterol |
| Bhatt  (2023, USA) | T: 18 (2)  C: 18 (1) | T: 16  C: 17 | T: Pegozafermin 27mg QW, SC  C: Placebo, SC | HDL-cholesterol | LDL-cholesterol |
| Charles  (2019, USA) | T: 24 (4)  C: 24 (2) | T: 20  C: 22 | T: Pegbelfermin (BMS-986036) 20 mg QD, SC  C: Placebo-matching with BMS-986036 QD, SC | HDL-cholesterol, ADP | Fasting glucose, insulin, triglycerides, LDL-cholesterol |
| Gaich  (2013, USA) | T: 15 (2-3)  C: 10 (2-3) | T: 12-13  C: 7-8 | T: LY2405319 20 mg QD, SC  C: Placebo-matching LY2405319 QD, SC | Triglycerides, total cholesterol, LDL-cholesterol, HDL-cholesterol, ADP | Fasting glucose, insulin, |
| Harrison  (2023, USA) | T: 20 (1)  C: 10 (1) | T: 20  C: 10 | T: Efruxifermin 50mg QW, SC  C: Placebo QW, SC | TG, ADP | - |
| Harrison  (2023, USA) | T: 43 (7)  C: 43 (1) | T: 35  C: 42 | T: Efruxifermin 50mg QW, SC  C: Placebo QW, SC | TG, LDL-cholesterol, HDL-cholesterol, ADP | - |
| Kaufman  (2020, USA) | T: 6 (0)  C: 9 (0) | T: 6  C: 9 | T: Efruxifermin (AKR-001) 70 mg QW, SC  C: placebo, SC | Fasting glucose, insulin, triglycerides, HDL-cholesterol | - |
| Kim  (2017, USA) | T: 21 (2)  C: 22 (3) | T: 19  C: 19 | T: PF-05231023 150 mg QW, IV  C: 0.9% W/V sodium chloride injection QW, USP | ADP | Fasting glucose, insulin, total cholesterol |
| Loomba  (2023, USA) | T: 73  C: 71 | T: 66  C: 61 | T: Pegozafermin 30mg QW, SC  C: Placebo, SC | Triglycerides, HDL-cholesterol, ADP | LDL-cholesterol, |
| Rader  (2022, USA) | T: 30 (7)  C: 31 (6) | T: 30  C: 31 | T: LLF580 300 mg every 4 weeks, SC  C: placebo, SC | Insulin, triglycerides, total cholesterol, LDL-cholesterol, HDL-cholesterol, ADP | Fasting glucose |
| Sanyal  (2018, USA) | T: 25 (1)  C: 26 (1) | T: 25  C: 26 | T: Pegbelfermin (BMS-986036) 10 mg QD, SC  C: placebo QD, SC | LDL-cholesterol, HDL-cholesterol | Triglycerides |

**Table S4** Detailed information of trials in this meta-analysis.

***Note:*** T, trial group; C, control group; QD, once a day; QW, once a week; Q4W, once every 4 weeks; W/V, weight/volume; IV, intravenous; SC, subcutaneous; USP, United States Pharmacopeia; LDL-cholesterol, low-density lipoprotein cholesterol; HDL-cholesterol, high-density lipoprotein cholesterol; ADP, adiponectin.**Table S5** The methodological quality of the included RCTs via the risk of bias.

| **Study**  **(year)** | **Selection bias** | |  | **Performance** |  | **Detection bias** |  | **Attrition bias** |  | **Reporting bias** | **Other bias** | **Study quality** |
| --- | --- | --- | --- | --- | --- | --- | --- | --- | --- | --- | --- | --- |
|  | Random sequence generation | Allocation concealment |  | Blinding of participants and personnel |  | Blinding of outcome assessment |  | Incomplete outcome data |  | Selective  Reporting |  |  |
| Abdelmalek (2024) | Low risk | Low risk |  | Low risk |  | Low risk |  | Low risk |  | Low risk | Unclear risk | Good |
| Bhatt (2023) | Unclear risk | Unclear risk |  | Low risk |  | Unclear risk |  | Low risk |  | Low risk | Unclear risk | Fair |
| Charles (2019) | Low risk | Unclear risk |  | Low risk |  | Unclear risk |  | Low risk |  | Low risk | Unclear risk | Good |
| Gaich (2013) | Unclear risk | Low risk |  | Low risk |  | Unclear risk |  | Low risk |  | Low risk | Unclear risk | Good |
| Harrison (2023) | Low risk | Low risk |  | Low risk |  | Low risk |  | Low risk |  | Low risk | Unclear risk | Good |
| Harrison (2023) | Low risk | Low risk |  | Low risk |  | Low risk |  | Low risk |  | Low risk | Unclear risk | Good |
| Kaufman (2020) | Low risk | Unclear risk |  | Low risk |  | Unclear risk |  | Low risk |  | Low risk | Unclear risk | Good |
| Kim (2017) | Unclear risk | Unclear risk |  | Low risk |  | Unclear risk |  | Low risk |  | Low risk | Unclear risk | Fair |
| Loomba (2023) | Low risk | Unclear risk |  | Low risk |  | Low risk |  | Low risk |  | Low risk | Unclear risk | Good |
| Rader (2022) | Low risk | Low risk |  | High risk |  | High risk |  | Low risk |  | Low risk | Unclear risk | Good |
| Sanyal (2018) | Low risk | Low risk |  | Low risk |  | Low risk |  | Low risk |  | Low risk | Unclear risk | Good |

| **Study (year)** | **Randomization** | **Allocation concealment** | **Blinding** | **Withdrawals and dropouts** | **Jadad Scores** |
| --- | --- | --- | --- | --- | --- |
| Abdelmalek (2024) | 2 | 2 | 2 | 1 | 7 |
| Bhatt (2023) | 1 | 0 | 2 | 1 | 4 |
| Charles (2019) | 2 | 0 | 2 | 1 | 5 |
| Gaich (2013) | 1 | 2 | 2 | 1 | 6 |
| Harrison (2023) | 2 | 2 | 2 | 1 | 7 |
| Harrison (2023) | 2 | 2 | 2 | 1 | 7 |
| Kaufman (2020) | 2 | 0 | 2 | 1 | 5 |
| Kim (2017) | 1 | 0 | 2 | 1 | 4 |
| Loomba (2023) | 2 | 0 | 2 | 1 | 5 |
| Rader (2022) | 2 | 2 | 0 | 1 | 5 |
| Sanyal (2018) | 2 | 2 | 2 | 1 | 7 |

**Table S6** The methodological quality of the included RCTs via the modified Jadad scale.

**Table S7** Safety summary.

| **Study (year)*** | **Abdelmalek** | |  | **Bhatt** | |  | **Charle** | |  | **Harrison** | |  | **Harrison** | |  | **Kaufman** | |  | **Kim** | |  | **Loomba** | |  | **Rader** | |  | **Sanyal** | |
| --- | --- | --- | --- | --- | --- | --- | --- | --- | --- | --- | --- | --- | --- | --- | --- | --- | --- | --- | --- | --- | --- | --- | --- | --- | --- | --- | --- | --- | --- |
|  | C | T |  | C | T |  | C | T |  | C | T |  | C | T |  | C | T |  | C | T |  | C | T |  | C | T |  | C | T |
| Sample size | 39 | 37 |  | 18 | 18 |  | 24 | 24 |  | 10 | 20 |  | 43 | 43 |  | 17 | 6 |  | 22 | 21 |  | 69 | 72 |  | 31 | 30 |  | 26 | 25 |
| Nausea | 5 | 11 |  | 0 | 2 |  | 0 | 1 |  | 2 | 9 |  | 10 | 18 |  | 0 | 2 |  | 1 | 2 |  | 6 | 23 |  | 2 | 18 |  | 2 | 4 |
| Diarrhea | 6 | 16 |  | 1 | 4 |  | 1 | 4 |  | 1 | 10 |  | 8 | 17 |  | 1 | 2 |  | 0 | 8 |  | 4 | 14 |  | 4 | 8 |  | 2 | 3 |
| Dyspepsia | - | - |  | - | - |  | 0 | 1 |  | - | - |  | - | - |  | 1 | 2 |  | - | - |  | - | - |  | 1 | 4 |  | - | - |
| Abdominal pain | - | - |  | 0 | 2 |  | - | - |  | 2 | 3 |  | - | - |  | 0 | 0 |  | - | - |  | 0 | 1 |  | - | - |  | - | - |
| Vomiting | - | - |  | 0 | 1 |  | 0 | 0 |  | 0 | 4 |  | 4 | 6 |  | 0 | 2 |  | 0 | 2 |  | 2 | 10 |  | 1 | 9 |  | - | - |
| Frequent bowel Movements | - | - |  | - | - |  | - | - |  | - | - |  | 1 | 0 |  | - | - |  | 0 | 1 |  | - | - |  | - | - |  | 0 | 5 |
| Injection site erythema | - | - |  | 0 | 2 |  | 1 | 2 |  | 0 | 5 |  | 8 | 8 |  | 0 | 0 |  | 1 | 3 |  | 3 | 11 |  | - | - |  | - | - |
| Injection site reaction  (bruising or Rash) | 4 | 7 |  | 0 | 1 |  | 5 | 1 |  | 1 | 10 |  | 3 | 10 |  | 0 | 2 |  | 1 | 0 |  | 1 | 7 |  | - | - |  | 0 | 2 |
| Increased appetite | - | - |  | - | - |  | 2 | 1 |  | - | - |  | 2 | 10 |  | 0 | 2 |  | - | - |  | 1 | 10 |  | 2 | 3 |  | - | - |
| Decreased appetite | - | - |  | - | - |  | - | - |  | - | - |  | - | - |  | - | - |  | - | - |  | - | - |  | 0 | 5 |  | - | - |
| Hunger | - | - |  | - | - |  | - | - |  | - | - |  |  |  |  | - | - |  | 0 | 2 |  | - | - |  | - | - |  | - | - |
| Headache | - | - |  | - | - |  | 2 | 0 |  | 1 | 4 |  | 5 | 5 |  | 4 | 0 |  | 1 | 2 |  | 5 | 6 |  | -- | - |  | 3 | 1 |
| Fatigue | - | - |  | - | - |  | 0 | 0 |  | - | - |  | - | - |  | - | - |  | - | - |  | - | - |  | - | - |  | 5 | 1 |
| Dizziness | - | - |  | - | - |  | - | - |  | - | - |  | - | - |  | - | - |  | 1 | 2 |  | - | - |  | - | - |  | - | - |
| Upper respiratory tract infection | - | - |  | - | - |  | 0 | 0 |  | - | - |  | - | - |  | 0 | 1 |  | 0 | 1 |  | - | - |  | 4 | 5 |  | - | - |
| Nasopharyngitis | - | - |  | - | - |  | 1 | 1 |  | 2 | 7 |  | - | - |  | - | - |  | - | - |  | - | - |  | - | - |  | - | - |
| Dermatitis contact | - | - |  | - | - |  | - | - |  | - | - |  | - | - |  | - | - |  | - | - |  | - | - |  | - | - |  | - | - |
| Cough | - | - |  | - | - |  | 2 | 0 |  | - | - |  | - | - |  | - | - |  | - | - |  | - | - |  | - | - |  | 2 | 1 |
| Urinary tract infection | - | - |  | - | - |  | - | - |  | - | - |  | - | - |  | - | - |  | - | - |  | 5 | 8 |  | - | - |  | 2 | 1 |
| Increased blood CPK | - | - |  | - | - |  | 0 | 0 |  | - | - |  | - | - |  | - | - |  | - | - |  | - | - |  | - | - |  | - | - |
| Palpitations | - | - |  | - | - |  | - | - |  | - | - |  | - | - |  | 0 | 2 |  | - | - |  | - | - |  | - | - |  | - | - |
| Back pain | - | - |  | - | - |  | - | - |  | - | - |  | - | - |  | 2 | 1 |  | - | - |  | - | - |  | 2 | 3 |  | - | - |
| Muscle spasms | - | - |  | - | - |  | - | - |  | - | - |  | - | - |  | 0 | 0 |  | - | - |  | 1 | 7 |  | 3 | 1 |  | - | - |
| Serious AEs | 3 | 7 |  | 0 | 0 |  | 1 | 1 |  | 1 | 0 |  | 0 | 4 |  | 0 | 0 |  | - | - |  | 3 | 3 |  | 1 | 2 |  | 1 | 1 |
| Discontinuation  due to AEs | 0 | 0 |  | 0 | 4 |  | 0 | 2 |  | 0 | 1 |  | 0 | 3 |  | 1 | 0 |  | - | - |  | 1 | 8 |  | 1 | 2 |  | 0 | 0 |
| All AEs | 35 | 35 |  | 9 | 14 |  | 13 | 14 |  | 8 | 19 |  | 38 | 42 |  | 12 | 5 |  | - | - |  | 47 | 61 |  | - | - |  | 15 | 18 |

***Note:*** *The study by Gaich did not provide detailed information about adverse events, which reported three cases of serious adverse events and three cases of withdrawal due to adverse events, with 94.5% of participants experiencing mild adverse events. T, trial group; C, control group; CPK, creatine phosphokinase; AE, adverse events; “-”, not reported.


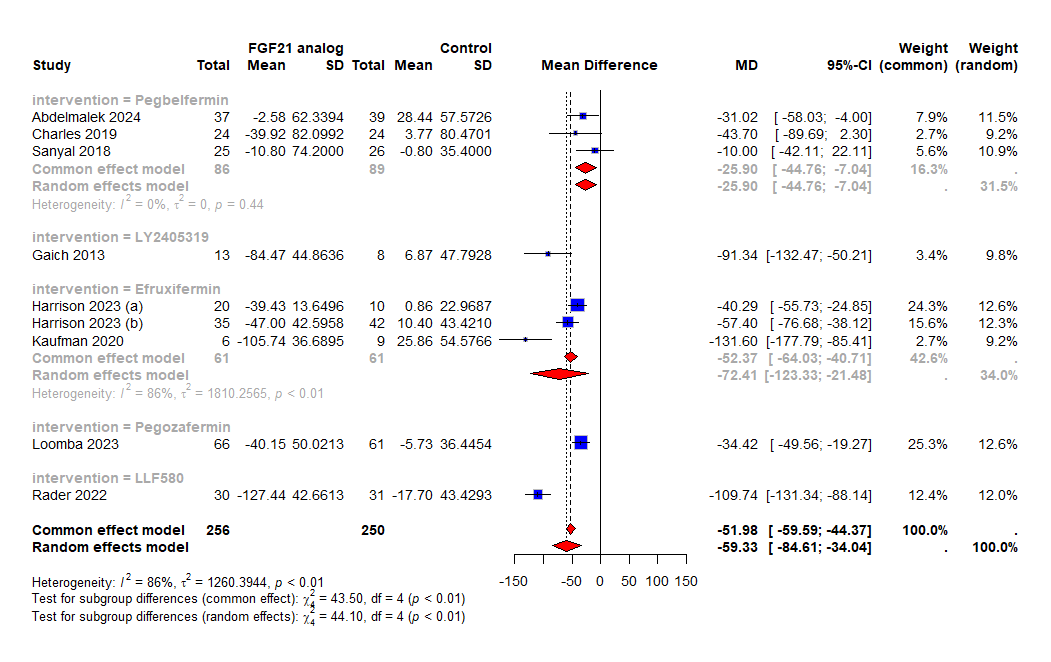


**Figure S1** Subgroup analyses for various FGF21 analogs in triglycerides. MD, mean difference.


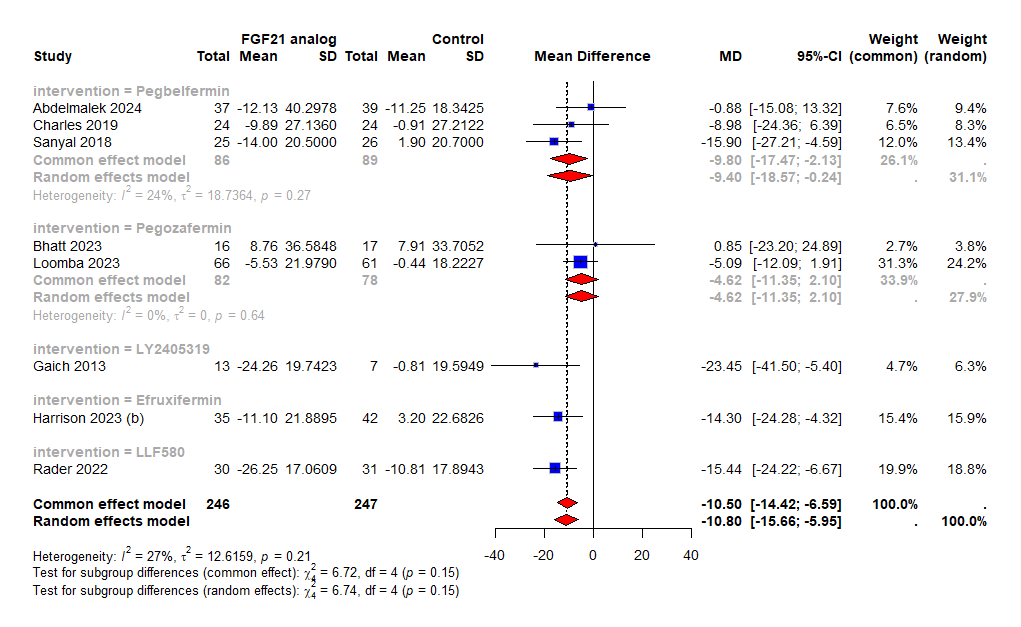


**Figure S2** Subgroup analyses for various FGF21 analogs in low-density lipoprotein cholesterol. MD, mean difference.

**
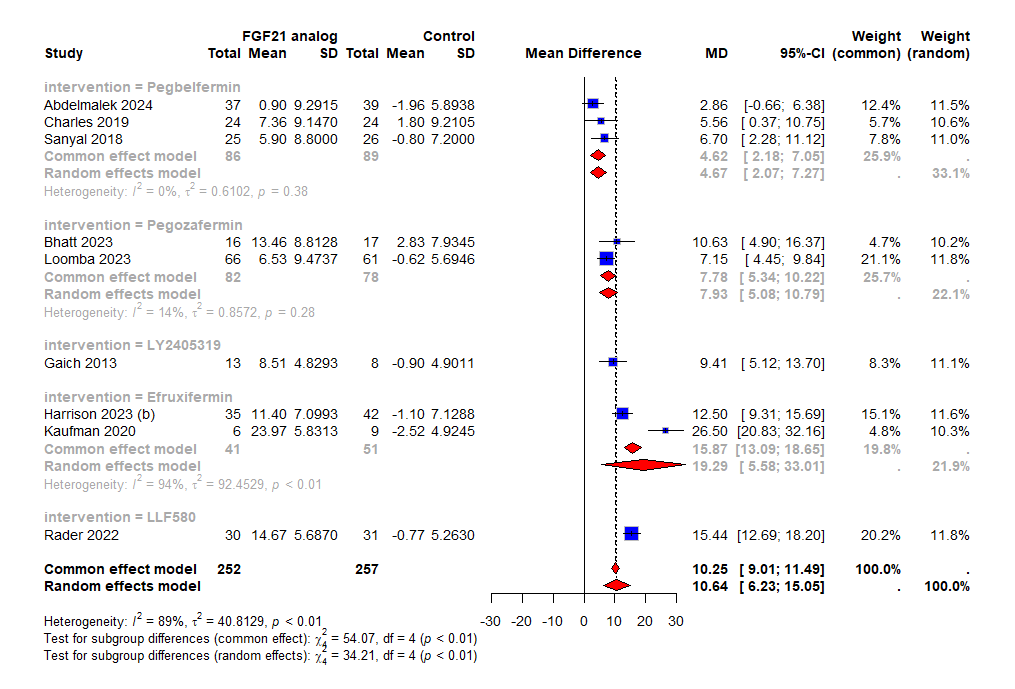
**

**Figure S3** Subgroup analyses for various FGF21 analogs in high-density lipoprotein cholesterol. MD, mean difference.


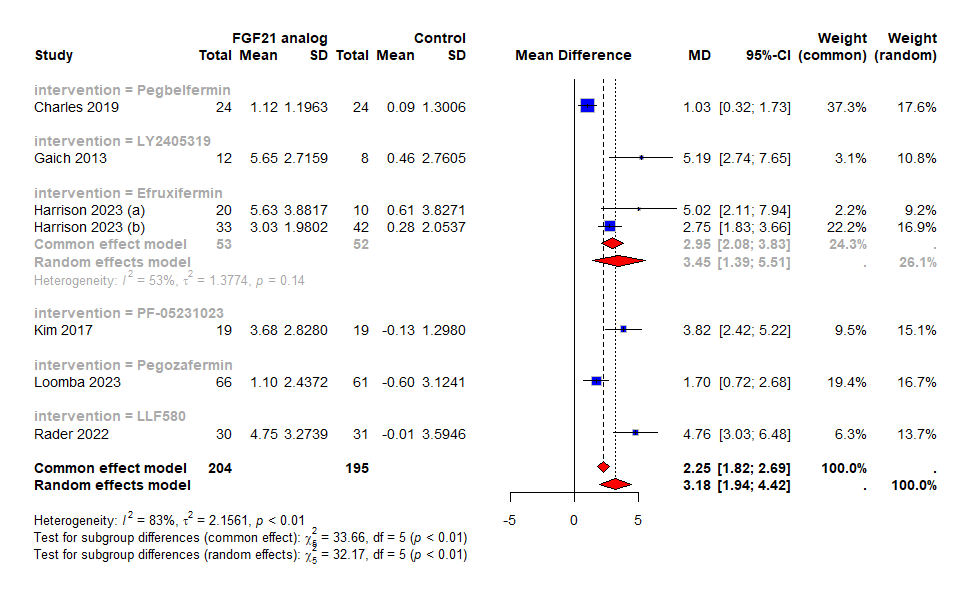


**Figure S4** Subgroup analyses for various FGF21 analogs in circulating adiponectin levels. MD, mean difference.


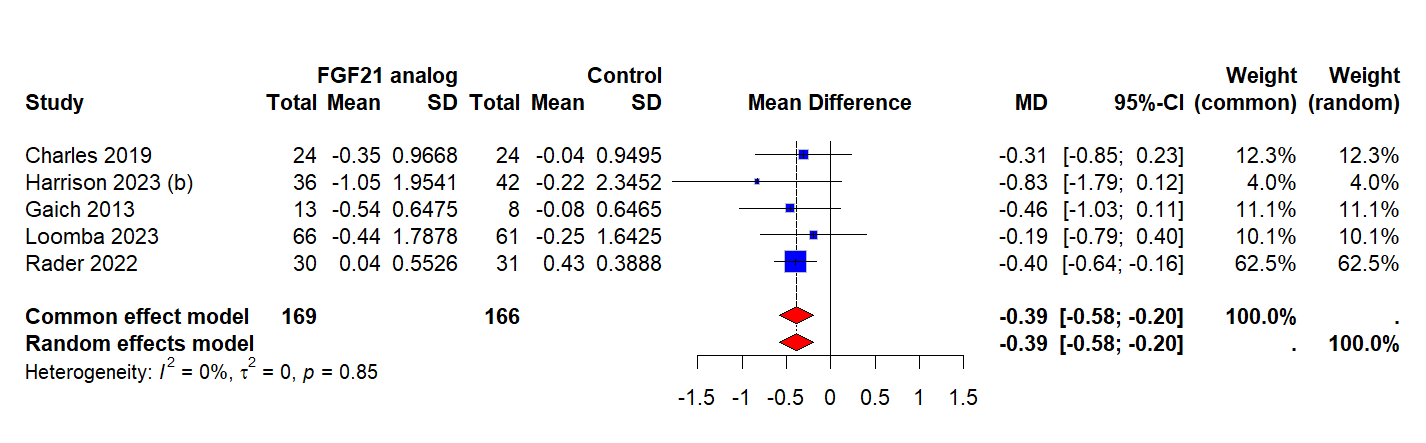


**Figure S5** Forest plots for changes after administrating FGF21 analogs in body mass index. MD, mean difference.


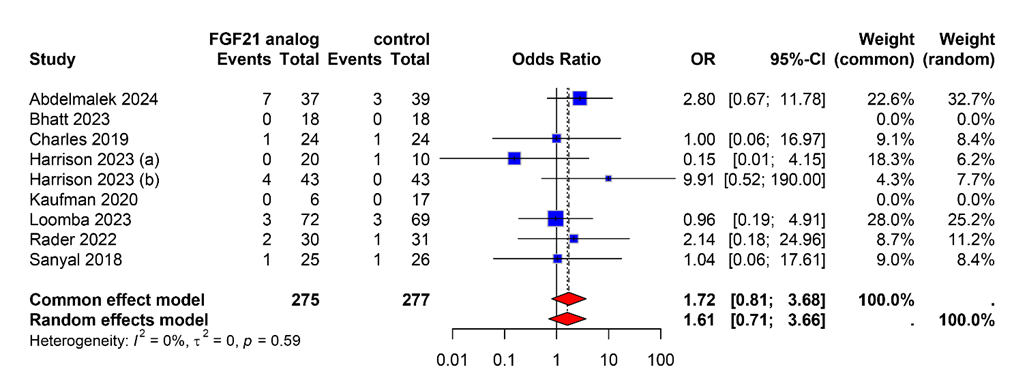


**Figure S6** Forest plot for incidence of serious adverse events after administrating FGF21 analogs. OR, odds ratio.


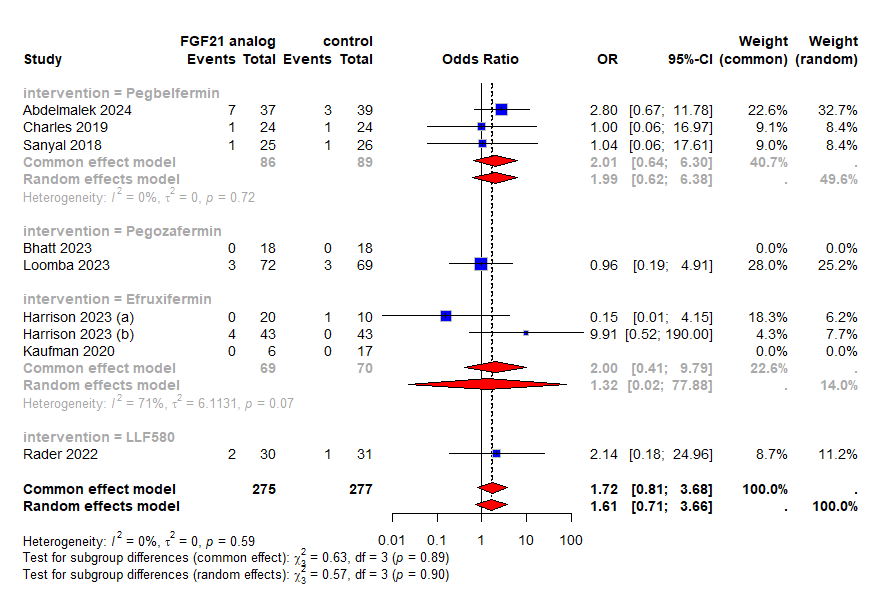


**Figure S7** Subgroup analyses for various FGF21 analogs in incidence of serious adverse events. OR, odds ratio.
